# Comment on “Predictors of Indoor Radon Concentrations in Pennsylvania, 1989–2013”

**DOI:** 10.1289/ehp.1510250

**Published:** 2015-11-01

**Authors:** Terry Engelder

**Affiliations:** Department of Geosciences, The Pennsylvania State University, University Park, Pennsylvania, USA

When dividing Pennsylvania counties into five groups, Casey et al. reported an upward trend in radon concentration between 2004 and 2012 in all groups. NBC News subsequently warned, “Rising levels of toxic gas found in homes near fracking sites” (NBC News 2015). This headline is misleading.

The county groups of most interest—high, low, and no Marcellus activity—show a difference in adjusted geometric mean indoor radon concentration of < 0.8 pCi/L during any given year. Because the counties with no drilling activity had higher indoor radon concentrations than those with low drilling activity, it is unreasonable to attach a physical meaning to the difference of < 0.4 pCi/L between high- and no-activity counties at any time during Marcellus development. This result (i.e., < 0.4 pCi/L) would not be expected if ambient radon gas was escaping in significant volume from Marcellus drill pads.

While it is fair to separate counties with high and low activity, both groups are part of the same geological province, the Appalachian Plateau. High- and low-activity counties are interspersed in a patchwork such that, prior to any Marcellus activity, the two data sets should have shown similar but not necessarily identical trends. Figure 4 of Casey et al. shows that with the exception of 1995, 1996, and 2000, they do exhibit the same up–down trends in radon, with predicted indoor radon concentrations in high-activity counties offset upward by a fraction of a pCi/L long before the arrival of drilling in Pennsylvania. The same trends carry through the years of Marcellus drilling. The no-activity counties are part of a different geological province, possibly giving rise to a different radon trend (Rodgers 1971), but even data from the no-activity counties demonstrate a similar up–down trend that carries through from before the arrival of Marcellus activity.

It is even more difficult to make the case that radon trends correlate with hydraulic fracturing, or fracking, if one considers the true arrival date of significant high-volume fracking in all but Washington County, Pennsylvania. Arguably, major drilling activities were not under way until the second half of 2008, and significant production of Marcellus gas was not under way until 2009 (PA DEP 2015). The authors state explicitly that “[o]nly unconventional wells (horizontal wells, hydraulic fracturing) were included” in their study. There was only one horizontal Marcellus well drilled in Pennsylvania in 2006 and only five by the end of 2007, all in Washington County (PA DEP 2015). Yet Figure 3A of Casey et al. indicates as many as 320 horizontal wells were drilled by the end of 2007, an obvious error in their paper. Figure 4 indicates radon concentration was trending upward in 2004 in all regions, long before fracking hit Pennsylvania. Prior to 2004, there is a clear down–up–down trend in all five study regions so that an up-trend after 2003 just mirrors a similar five-year cycle starting about 1994. Even implying a link between Marcellus activity and radon as much as 12.5 miles from the nearest drill pad unreasonably stretches any message found in the data.

The only way significant amounts of natural gas–related radon will enter homes at distances outside well-pad setbacks is through use of gas for heating or cooking. The cities on gas service should be most affected; in my experience, most rural homes are not connected with natural gas service. With heat turned off in summertime, the most common entry point for radon is, to my knowledge, through gas stoves on the first floor, which consume a small fraction of the natural gas burned in basement furnaces during the winter heating season. Yet, statistics from the Pennsylvania Department of Environmental Protection show that summertime radon concentrations are lower in both basements and first floors (Robert Lewis, personal communication, 14 May 2015), the latter with open windows being the logical entry point for direct diffusion from nearby pads. Throughout the year, first-floor radon concentration consistently remains at about half the basement concentration (Robert Lewis, personal communication, 14 May 2015). This is the behavior expected if radon enters from soil and migrates to the first floor from the basement by the stack effect.

Because up–down trends in radon were present in Pennsylvania prior to the arrival of Marcellus drilling, another explanation is necessary. Something as simple as soil moisture could account for the variation of radon with time. Trends that are similar back to 1989 suggest that the arrival of drilling activity is just a confounding factor making it more difficult to identify the real cause of the decadal-scale up–down trend in radon throughout Pennsylvania.

In sum, Casey et al. raised an alarm without justification. The literature shows that such alarms stress communities near drilling activity (Ferrar et al. 2013). Thus, it behooves health educators to be circumspect before placing statements in the peer-reviewed literature that can be manipulated by the media to cause public fear and concomitant stress-related health symptoms.
